# Thermal-induced force release in oxyhemoglobin

**DOI:** 10.1038/srep13064

**Published:** 2015-08-17

**Authors:** S. G. Gevorkian, A. E. Allahverdyan, D. S. Gevorgyan, Chin-Kun Hu

**Affiliations:** 1 Institute of Physics, Academia Sinica, Nankang, Taipei 11529, Taiwan; 2 Laboratoire de Physique Statistique et Systèmes Complexes, ISMANS, 44 ave. Bartholdi, 72000 Le Mans, France; 3 Yerevan Physics Institute, Alikhanian Brothers St. 2, Yerevan 375036, Armenia; 4 Institute of Fine Organic Chemistry, 26 Azatutian ave., Yerevan 0014, Armenia; 5 National Center for Theoretical Sciences, National Tsing Hua University, Hsinchu 30013, Taiwan

## Abstract

Oxygen is released to living tissues via conformational changes of hemoglobin from R-state (oxyhemoglobin) to T-state (desoxyhemoglobin). The detailed mechanism of this process is not yet fully understood. We have carried out micromechanical experiments on oxyhemoglobin crystals to determine the behavior of the Young’s modulus and the internal friction for temperatures between 20 °C and 70 °C. We have found that around 49 °C oxyhemoglobin crystal samples undergo a sudden and strong increase of their Young’s modulus, accompanied by a sudden decrease of the internal friction. This sudden mechanical change (and the ensuing force release) takes place in a partially unfolded state and precedes the full denaturation transition at higher temperatures. After this transformation, the hemoglobin crystals have the same mechanical properties as their initial state at room temperatures. We conjecture that it can be relevant for explaining the oxygen-releasing function of native oxyhemoglobin when the temperature is increased, e.g. due to active sport. The effect is specific for the quaternary structure of hemoglobin, and is absent for myoglobin with only one peptide sequence.

Since its discovery in 1840, the hemoglobin is one of the most extensively studied proteins^1^,^2^. This is related to its important physiological function: it carries oxygen from the lungs throughout the body allowing us to breathe and live. It consists of four globular units linked into a double-dimer tetrameric structure^1^,^2^ as shown schematically in Fig. 1. Each unit can carry one oxygen molecule O_2_ attached to its heme group. The hemoglobin structure is adapted to the needs of its function. First, its oxygen binding is cooperative: the response of hemoglobin with respect to O_2_ concentration has a S-shaped region, thereby a relatively slight decrease of the oxygen concentration between the lungs and the body brings in a significant decrease of the bound oxygen (up to 25%)^1^,^2^. Second, the oxygen binding ability decreases upon reducing the pH factor or increasing the concentration of CO_2_^3^. Due to this Bohr’s effect^3^ a tissue with a stronger need of oxygen receives it more. Cooperative oxygen unbinding of hemoglobin is explained by the change of its tetrameric conformational structure induced by binding of the first oxygen molecule^2^; see Fig. 1. The conformational states with (R-state or oxyhemoglobin) and without (T-state or desoxyhemoglobin) the four oxygen molecules are clearly distinguishable by their shape, as seen via X-ray crystallography^1^,^2^. *In vivo* those states are more like dynamic ensembles than fixed conformations^4^.

Many aspects of hemoglobin are well understood by now^2^. However, the physics of conformational changes and their interaction with external factors (pressure, oxygen concentration, temperature) is still under active scrutiny^5^,^6^,^7^,^8^. In particular, this concerns the thermal response of hemoglobin that is traditionally studied via denaturation experiments. It is known that the multidomain hemoglobin does not unfold via a single transition^5^. Rather, there is a wide transition zone ≃40°–60 °C that includes several events^5^. These events are typically interpreted as unfolding and/or internal aggregation, two standard mechanisms normally applied for describing thermal responses of multi-subunit proteins^5^,^6^.

Here we report the results of micromechanical experiments carried out on crystals of horse and human hemoglobin. We show that in its partially unfolded state—i.e. for a temperature higher than the physiological temperatures, but lower than the unfolding temperature—the hemoglobin responds to heating by a sudden release of force and a subsequent rapid increase of the Young’s modulus, which is similar to the finding reported by Ansari *et al*.^9^. The detailed structure of this effect is different for human and horse hemoglobin. We argue that the effect relates to certain slowly relaxing degrees of freedom of the quaternary structure that accumulate energy during heating and then suddenly release it at a well-defined temperature that is specific for hemoglobin.

Such effect is absent in the thermal response of myoglobin. This is a single-unit globular protein that displays a visible transition towards a partially denaturated state around a certain unfolding temperature^5^. Myoglobin also binds and unbinds oxygen, but does so without a sizable cooperativity. This relates to its function: myoglobin is a depot (not transporter) of oxygen in muscles. The difference between hemoglobin and myoglobin was also observed in heating and re-cooling data of Young’s modulus for two systems to be reported below.

The force-release effect reported here for hemoglobin crystals has several predecessors in biopolymer physics. Ansari *et al*. found an indirect experimental evidence that the low-temperature ligand unbinding of myoglobin is modulated by a sudden release of energy accumulated due to the ligand binding^9^. They proposed the term *proteinquake* for such effects. Later on, it was found computationally that proteinquakes are relevant to the functioning of myosin^10^ and adenylate kinase^11^. More specifically for hemoglobin, it is known that when crystals of deoxyhemoglobin are exposed to oxygen, they shatter due to the force released during the conformational transition from deoxy to oxyhemoglobin^12^,^13^. Postponing detailed connections with literature till the end of this paper, we stress already here that the presented report seems to be the first one, where a force-release effect was found under heating which is generally supposed to diminish mechanical features of biopolymers.

Note in this context that the advantage of using biopolymer crystals for measurement is that there is a possibility of controlling and displaying—via intermolecular contacts regulated by the crystal syngony and the water content—those motions of the macromolecule that can have only transient character in the solution^14^,^15^. Almost all the basic information on the hemoglobin structure came from experiments on soild-state hemoglobins. Figure 2 illustrates the structure of the hemoglobin crystal^1^,^2^,^16^,^17^. The solid state hemoglobin is closer to its *in vivo* state in mammal erythrocytes, where it is densely packed with concentration ≃34%^18^. It should be noted in this context that the orientation of hemoglobin molecules in erythrocytes is not random. First indications of this fact were obtained some 30 years ago^19^, but were not widely disseminated by that time. More recently this fact was confirmed by several independent studies; see e.g. Ref. 20.

## Results

**1.** We start with the denaturation curve of the human oxyhemoglobin at relative humidity 95% as shown in Fig. 3. The Young’s modulus *E* is stable between 25 °C and 36 °C and then decreases in discrete steps. In between of each step *E* is constant. We argue below that that these discrete steps are related to partial dissociation of the quaternary structure. The measured internal friction *θ* increases, because the structure breaking liberates new degrees of freedom that are able to dissipate the energy of forced oscillations; see Fig. 3.

But we could not perform the experiment for temperatures higher than 49 °C, because (at the employed excitation frequency ≃5 kHz of the plate oscillations) the crystals clove to pieces. The measurement of *θ* had to be terminated even earlier due to instability of results; see Fig. 3. The same breaking effect at 49 °C was observed for crystals of horse hemoglobin prepared under the same relative humidity 95%. This effect indicates the conformational changes taking place in the hemoglobin macromolecule. Recall in this context that deoxyhemoglobin crystals shatter after exposure to oxygen, since they cannot support a large conformational change related to the transition from deoxy to oxyhemoglobin^12^,^13^. The difference with our situation is that we work with oxyhemoglobin and that with us the crystals shatter after heating.

**Figure 2:**
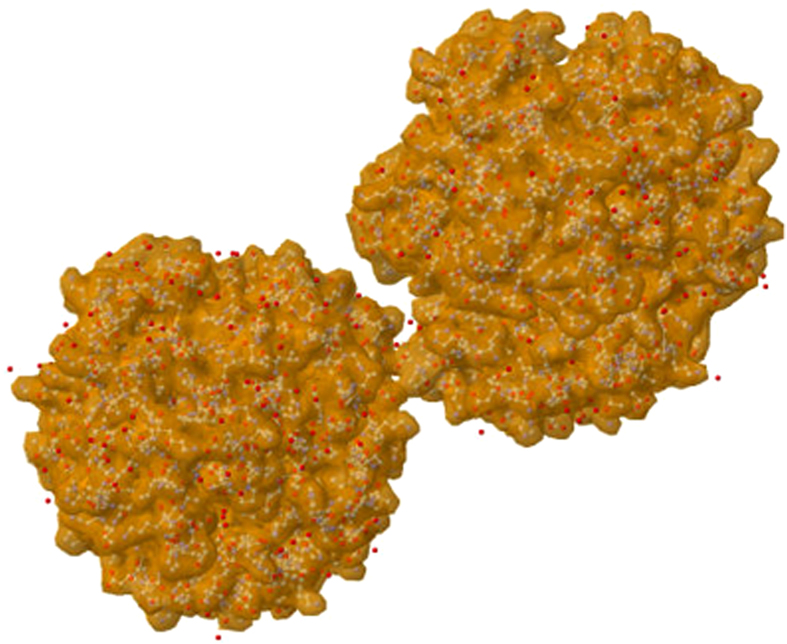
Contacts between two deoxyhemoglobin molecules in monoclinic crystal at resolution 1.74 Å with parameters P(1 2_1_ 1), *a* = 63.15 Å, *b* = 83.59 Å, *c* = 53.80 Å, *α* = 90.0°, *β* = 99.3°, *γ* = 90.0°^16^,^17^. Red points represent water molecules from the first Langmuir adsorption layer. The relative humidity is 95%–98%.

**Figure 3:**
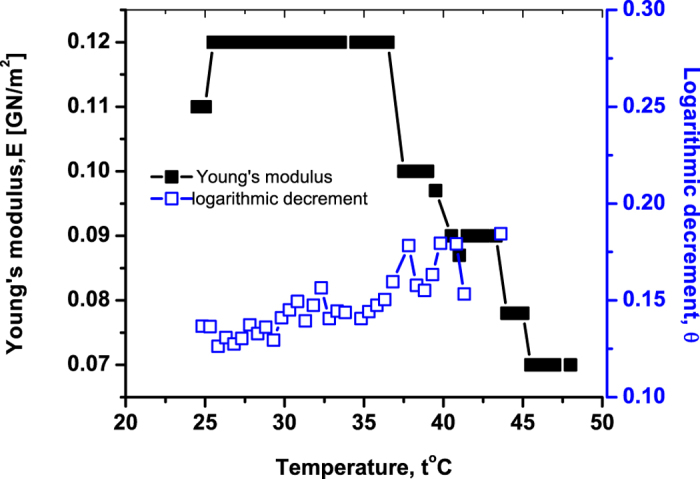
The Young’s modulus and the logarithmic decrement of damping of the human hemoglobin under heating. The relative humidity is now 95%.

**Figure 4:**
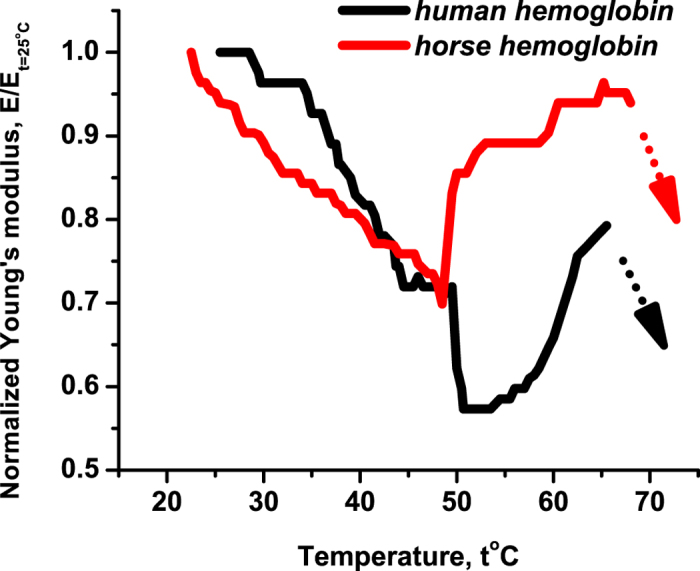
The Young’s modulus of the horse versus human hemoglobin. Both samples were prepared at the same initial temperature *t* = 25° and relative humidity 75%. The Young’s modulus at temperature *t* = 25°: *E*_*t*=25°_ = 0.75 GN/m^2^ for the horse hemoglobin sample and *E*_*t*=25°_ = 0.94 GN/m^2^ for the human sample.

**2.** To understand this effect, we equilibrated our samples under lower relative humidity of 75% at 25 °C. This was expected to prevent strong instabilities in the crystal^21^, since inter-molecular contacts in the crystal get stronger due to less inter-molecular water (Fig. 2). Figure 4 displays the behavior of the Young’s modulus for the human and horse hemoglobin under heating at relative humidity 75%. Note that compared to the 95%-humidity, the absolute value of the Young’s modulus increased for about 8 times; cf Fig. 4 with Fig. 3.

For temperatures from 20 °C to 49 °C the Young’s modulus *E* of the human hemoglobin decreases again in discrete steps. However, due to less inter-molecular water these steps are now shorter. Indeed, consider the temperature interval 25°–36 °C. For 95%-humidity *E* is constant there [see Fig. 3], but for 75%-humidity it still makes one sudden change in this interval; see Fig. 4. Hence the inter-molecular water does stabilize the hemoglobin structure against dissociation, though it decreases the absolute value of *E*. For horse hemoglobin the decay of *E* for temperatures from 20 °C till 49 °C is more gradual, but the stepwise change is still visible; see Fig. 4.

**Figure 5:**
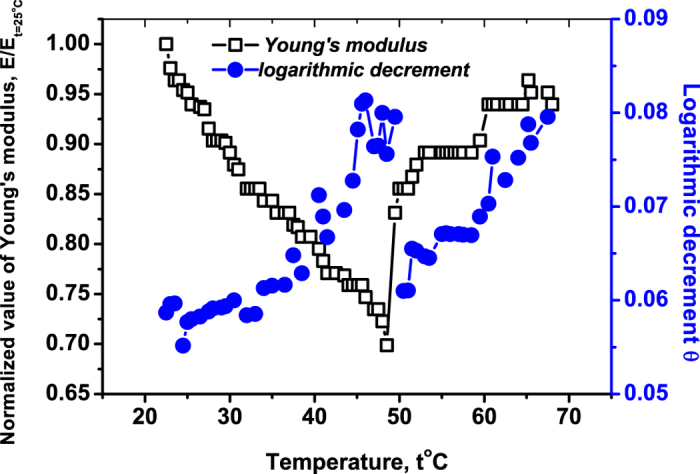
The normalized Young’s modulus *E*/*E*_*t*=25°_ and the logarithmic decrement of damping, *θ*, versus temperature *t* for monoclinic crystals of horse hemoglobin. The relative humidity is 75%. The heating rate is 0.1 °C/min and *E*_*t*=25°_ = 0.75 GN/m^2^.

**Figure 6:**
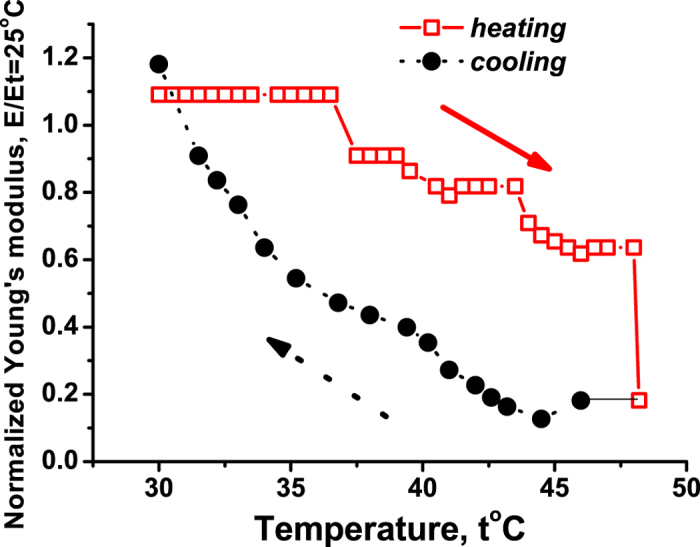
The Young’s modulus of the human hemoglobin under heating and re-cooling in the vicinity of 49 °C. The relative humidity is 95%. The heating rate is 0.1 °C/min. Temperature is smaller than 49 °C and *E*_*t*=25°_ = 0.11 GN/m^2^.

**Figure 7:**
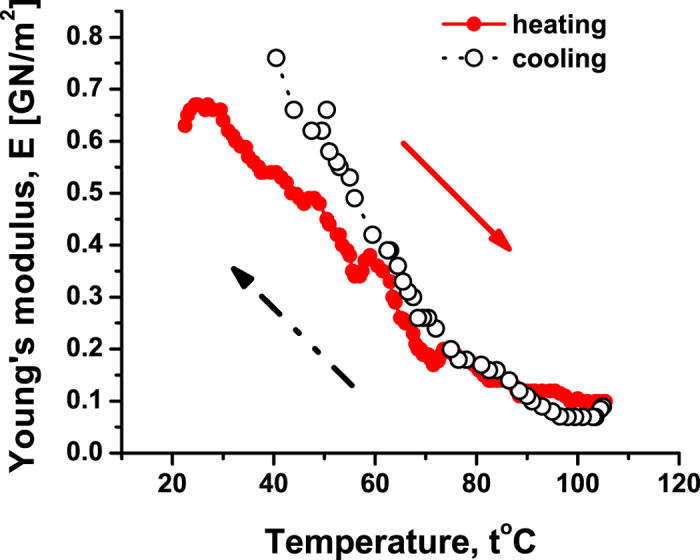
The Young’s modulus *E* versus temperature *t* under heating and recooling for monoclinic crystals of sperm-whale myoglobin. The denaturation process is seen to be approximately reversible.

**Figure 8:**
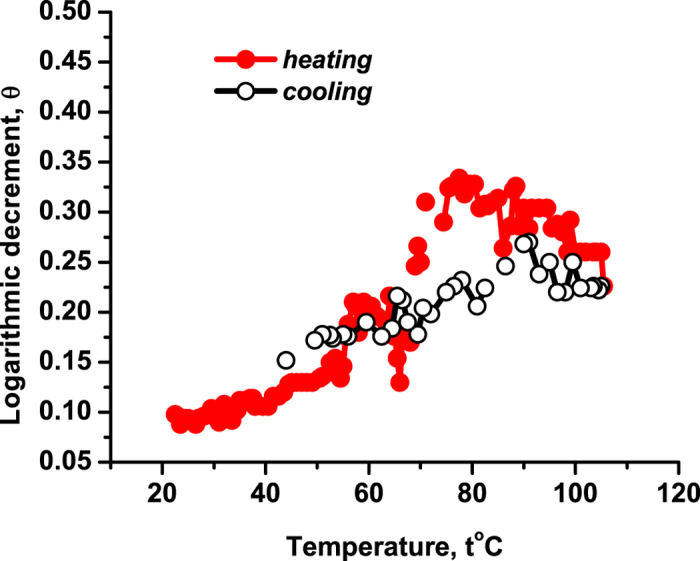
The logarithmic decrement of damping, *θ* versus temperature *t* under heating and recooling for monoclinic crystals of sperm-whale myoglobin.

**3.** But the temperature 49 °C is again a special one both for the human and horse hemoglobin; see Figs 4, 5, 6. In its vicinity, the Young’s modulus *E* changes *abruptly*. We prescribe these effects to the quaternary structure of hemoglobin, since myoglobin which lacks this structure, but still has well-defined tertiary and secondary structure does not show this effect [see Figs 7 and 8 below]. Thus, it is plausible that certain degrees of freedom of the quaternary structure have long relaxation times. During gradual heating, they go out of equilibrium, accumulate energy in elastic deformations and then suddenly release this strain energy at 49 °C.

The abrupt change of the Young’s modulus for relative humidity 75% is clearly the same effect that is responsible for breaking the crystals at a higher relative humidity 95%, where inter-molecular contacts are weaker [see **1**]. However, for the human hemoglobin *E* abruptly *decreases* at 49 °C, while for the horse hemoglobin it abruptly *increases*. We see that the precise type of the mechanical event—i.e., whether its Young’s modulus increases or decreases—depends on the type of hemoglobin (human or horse), but the temperature of the event is to a larger extent independent from the type, as Fig. 4 shows [we come back to this difference in **4**]. It is also independent from the relative humidity, in contrast to the absolute value of *E* and the pattern of its change. Recall that hemoglobin crystals of human and horse have the same crystal structure (monoclinic), but the space groups and lattice sizes for these situations are different^22^,^23^; see **Materials** for more details.

Figure 5 presents the behavior of both *θ* and *E* for the horse hemoglobin. It is seen that in the immediate vicinity of 49 °C, *θ* abruptly decreases basically to the value it had at ≃35 °C. This indicates that the degrees of freedom liberated during the previous melting stage get blocked again.

The evolution of *E* and *θ* under heating with temperatures higher than 50 °C is different: *θ* starts to grow again indicating a new trend in structure breaking; see Fig. 5. But *E* gradually increases for both human and horse hemoglobin; see Fig. 4. We interpret this effect via the intra-tetrameric aggregation of partially denaturated monomers of hemoglobin, because such increase of *E* is absent for the monomeric myoglobin; see Fig. 7 below.

It is impossible to prescribe the event at 49 °C to the aggregation *only*, because the changes of *E* and *θ* take place within a too narrow temperature interval. We also cannot prescribe this effect to unfolding, since the decreasing *θ* indicates the ordering (rather than disordering) of certain degrees of freedom. Apparently, the only possibility left is that the event at 49 °C indicates the transition of the partially denaturated tetramer from the R-state to another conformational state with different visco-elastic features.

Figure 6 displays the heating-recooling dynamics of the human hemoglobin at relative humidity 95% around 49 °C. The effect is irreversible, but the initial value of *E* is roughly recovered for 30 °C, albeit the characteristic stepwise pattern of decreasing *E* under heating is not seen during the recooling. This indicates that the discrete steps relate to dissociation of the hemoglobin quaternary structure, which (as compared to the tertiary and secondary structures) is expected to be the most fragile one. Figure 6 confirms that the event at 49 °C takes place in a partially unfolded state.

**4.** We now face a non-trivial situation: the event happens at the same temperature 49 °C for both horse and human hemoglobin. But, the behavior of the Young’s modulus is very different: it suddenly increases for the horse hemoglobin (more rigid structure for higher temperatures), but decreases for human hemoglobin (less rigid). We repeated the experiment with several different samples, to be sure that the difference between human and horse hemoglobin is well reproduced.

It is well known that these two macromolecules differ by 20–25 amino acid residues on each hemoglobin unit^24^. Due to such differences the horse hemoglobin has lower reactivity with respect to certain cofactors regulating oxygen binding^25^.

We conjecture that these biochemical differences are reflected in the mechanical features of hemoglobin around 49 °C. The slow transformations that are responsible for the mechanical event at 49 °C are most probably the same for the horse and human hemoglobin, otherwise the temperature 49 °C could not be the same for both situations. But since the environments of these degrees of freedom in horse and human hemoglobin are different, we get a different overall response for horse and human situations.

**5.** For temperatures higher than 65 °C the Young’s modulus for the hemoglobin crystals [both for human and horse] abruptly decreases again (not shown on figures) indicating on its full denaturation. For such high temperatures we expect that even the secondary structure of the macromolecule is broken. Our experimental samples became unstable at temperatures higher than 70 °C, so that no reliable data could be extracted.

**6.** To gain more evidence on whether the described effect is specific for the tetrameric structure of hemoglobin, we performed the same experiment with the myoglobin crystals and showed the result in Fig. 7. Upon heating, the Young’s modulus *E* of the myoglobin decreases *gradually*, without discrete steps. This is consistent with breaking the tertiary structure of the myoglobin towards acquiring a more labile state. This also confirms that the discrete steps seen in Figs 3 and 4 relate to the dissociation of the quaternary structure, which is absent for myoglobin. Apart from relatively small non-monotonicity of *E* around 57 °C, the behavior of *E* is monotonic and reversible upon recooling. It is seen though that the reversibility is more visible for *T* > 57 °C than for lower temperatures, since at low temperatures *E* is more influenced by the well-defined myoglobin tertiary structure, which is not completely recovered after recooling. It is known that the calorimetric experiments for myoglobin show a well-displayed transition to a partially denaturated (molten) state, where its tertiary structure is partially lost; see^5^ for further references. Depending on certain experimental conditions this happens around 60°–80 °C^5^. But the mechanical features of myoglobin do not change suddenly during this transition to the partially molten state. This corresponds with the behavior of the logarithmic decrement of damping for the myoglobin—see Fig. 8— and is consistent with the gradual decrease of *E* with temperature; see Fig. 7.

### Relations to previous work

Several experimental studies were carried out via various methods (calorimetry, optics) on liquid-state samples displayed that something peculiar happens with hemoglobin around 49 °C^5^,^6^,^7^. The authors of^5^ and^6^ prescribed the 49 °C event to the onset of aggregation. As we saw, this is correct, but essentially incomplete: the aggregation indeed starts around 49 °C, but there is certainly more there than simply aggregation. Artmann *et al*. found indications of conformational transitions (not reducible to aggregation) at 49 °C using optical methods on liquid state human hemoglobin^7^. This is close to our results. They extensively studied hemoglobin of other species and noted that the event correlates with the physiological temperature and relates to the motion of erythrocytes (red blood cells)^7^. They also suggested that the scenario of this conformational transition can be similar to the glass transition in polymers^7^. Also this suggestion is confirmed by our results, because the peak of the internal friction around transition temperature [see Fig. 5] is a known indication of the glass transition, as was employed recently for detecting the glass transition in collagen^26^,^27^; see also Ref. 15,28, 29, 30, 31 in the context of glassy features of biopolymers.

Several decades ago it was conjectured that conformational changes related to biopolymer functioning proceed via mechanical motion of certain mesoscopic degrees of freedom^32^. This *protein-machine* conjecture was discussed in^9^, where certain aspects of the low-temperature kinetics of myoglobin were interpreted via proteinquakes: a sudden release of energy accumulated in elastic degrees of freedom. Later on, proteinquakes were found to be relevant for functioning of myosin^10^ and adenylate kinase^11^. An important aspect of this research was that proteinquakes were connected to partial unfolding of the biopolymer tertiary structure^10^,^11^. This agrees with our finding that the heating-induced force release at 49 °C takes place in a partially unfolded state of hemoglobin.

Meanwhile the protein-machine conjecture was supported from another angle: not only the protein functioning resembles that of a machine, but also the performance of an optimal (high efficiency and a large power) heat engine—as described by a generalized Carnot cycle—has shown deep analogies with protein physics and the folding-unfolding transition^33^.

## Discussion

Our main message is that upon temperature elevation horse and human hemoglobins experience a conformational transition around 49 °C detectable via suddenly changing Young’s modulus and decreasing internal friction. We argued that this is a mechanical event and that it cannot be traced back to denaturation and/or aggregation. The precise scenario of the event—but not its temperature—appears to be dependent on the type of hemoglobin. We conjectured that this difference relates to structural differences in the horse and human hemoglobin.

The message of our results for the hemoglobin functioning is that its mechanical features can be triggered by temperature *in addition* to other pertinent factors such as pH or oxygen concentration. In particular, our results can turn out to be relevant for understanding the process of oxygen release, because in tissues the oxygen release should take place much faster than the oxygen consumption in lungs.

Note that the global temperatures ≥42 °C are lethal for humans and horses. However, the *local* temperature can easily go to values higher than 42 °C without causing any serious damage to living tissues. A pertinent example is the hyperthermia treatment of cancer, where the local temperature goes to values higher than 45–48 °C without destroying healthy tissues. Recall as well that the orientation of hemoglobin molecules in erythrocytes is not random (in that respect they are similar to protein crystals)^19^,^20^.

In conclusion, we reiterate that the main experimental result of this work—force reease in heated hemoglobin—can be interpreted as a sudden, temperature-controlled mechanic motion. The mechanic character of this motion is to be compared with the fact that certain features of proteins may well have glassy properties. Recall that the glassy state is yet another (different from a mechanic motion) form of non-equilibrium that is characterized by slow relaxation and strong memory effects^34^,^35^,^36^. By now there are many experimental and numerical indications of such a state in biopolymers^15^,^26^,^27^,^28^,^29^,^30^,^31^. It should be interesting to carry out a direct experiments and study in which specific way the glassy state in proteins coexists with the mechanic motion.

## Methods

### Materials

Monoclinic crystals of horse and human hemoglobin were grown following the modified method of Drabkin^37^,^38^,^39^. Drabkin uncovered that hemoglobin crystals vary from one species to another, e.g. they are different for dog, horse and human. He also showed that hemoglobin crystalizes in three systems (syngonies): monoclinic, rhombic and hexagonal^37^,^38^,^39^. For our micro-mechanical experiments only the monoclinic is suitable, because we need pinacoid crystals samples.

Our solution contained 33 mg/ml oxyhemoglobin, 1% sodium oxalate, 0.9 M potassium phosphate and had pH = 6.5. It was poured on glass weighing cups, which were put inside the crystallization chamber. At the bottom of the chamber we poured a solution of 3% sodium oxalate and 2.7 M potassium phosphate (pH = 6.5). Crystallization was processed under 4–6 °C for several months. The crystals came out as thin rhomboids with the length around *L* = 4 mm, width *b* = 0.05 mm and thickness *h* = 0.005 mm (we stress that different crystal samples have slightly different values of *L*, *b* and *h*, what are given here are the characteristic values). This form did not differ from that described in Ref. 37 where the crystallization was processed in the presence of sodium oxalate and (NH_4_)_2_SO_4_. The obtained crystals can be utilized directly (without fixation) for our micromechanical experiments. A typical structure is shown in Fig. 2^16^,^17^. Monoclinic crystals of sperm-whale myoglobin were grown following the method of Ref. 40.

Note that hemoglobin crystals of human and horse have the same crystal structure (monoclinic), but the space groups and lattice sizes for these situations are different. Here we briefly recall the X-ray diffraction data^22^,^23^ obtained with resolution of ≈2 Å. For horse hemoglobin the space group is C222_1_, while the unit cell parameters (in Å): *a* = 76.96, *b* = 81.70, *c* = 92.63^22^. For human hemoglobin the space group is P2_1_2_1_2A, and the unit cell parameters are *a* = 97.05, *b* = 99.50, *c* = 66.11^23^. The horse and human hemoglobin crystals also differ by their salt content.

### Experimental methods

The dynamic Young’s modulus *E* and the logarithmic decrement of damping *θ* for hemoglobin and myoglobin crystals were studied via the method described in^15^,^41^. The method is based on analyzing electrically induced transverse mechanical oscillations of the plate which is fixed by a cantilever. The dynamic Young’s modulus measures the elasticity degree. To measure *E*, one changes smoothly the frequency *f* of the induced oscillations to determine experimentally the basic resonance frequency *f*_0_, which corresponds to the maximal oscillation amplitude of plate’s loose end. The Young’s modulus for the long axial direction of the plate is given as^42^
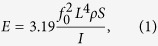
where *L* is the length of sample, *S* = *bh* is the cross-section area of the plate, *ρ* is its density, and *I* = *bh*^3^/12 is the main inertia moment of the plate.

The logarithmic decrement of damping *θ* is used as a measure of the internal friction, and is defined as 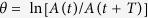, where *A*(*t*) is the oscillation amplitude in time *t*, and *T* is the period. *θ* is measurable by two related methods. One can measure the length of the resonance curve and calculate *θ* according to: 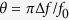, where Δ*f* is the difference of frequencies between the oscillation amplitude at the maximum amplitude and 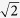 times less than the maximum. A more precise determination method amounts to measuring the phase shifts between the oscillations of exciting force and the sample loose end^15^,^41^.

## Additional Information

**How to cite this article**: Gevorkian, S. G. *et al*. Thermal-induced force release in oxyhemoglobin. *Sci. Rep*. **5**, 13064; doi: 10.1038/srep13064 (2015).

## Figures and Tables

**Figure 1:**
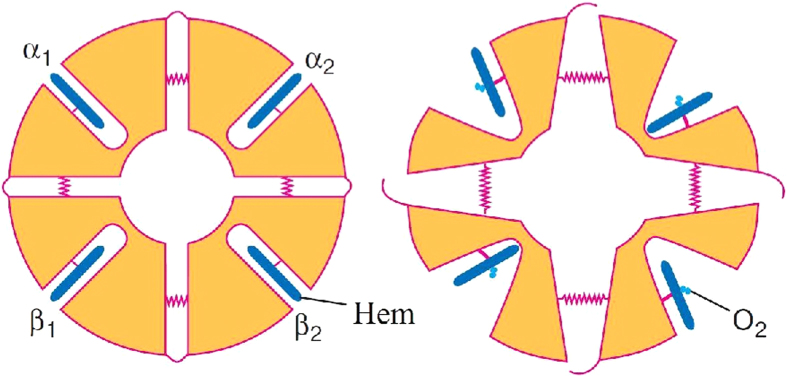
Schematic representation of the hemoglobin quaternary structure with four globular sub-units (yellow color: *α*_1_, *α*_2_, *β*_1_, *β*_2_) and four heme groups (blue color). (Left) T-form, where the access to the oxygen binding sites of the heme groups is restricted and the overall structure is more compact; (Right) R-form with easier access to the heme groups by oxygen molecule O_2_ and a more loose structure. The cooperativity of hemoglobin during oxygen binding is related to the transition from the T form to the R form.
